# Seed quality and relative lignan profiles of sesame prospected from northern Ghana

**DOI:** 10.1016/j.heliyon.2024.e39108

**Published:** 2024-10-09

**Authors:** Henry Ofosuhene Sintim

**Affiliations:** Institute of Applied Science and Technology, College of Basic and Applied Sciences, University of Ghana, Legon, Ghana

**Keywords:** Sesame, Seed quality, Pericarp colour, Viability, Lignans

## Abstract

The sesame seed contains oil, protein, dietary fibre, and several minerals and it is also a store of lignans. Lignans are key selection factors for sesame quality due to their health, nutritive and market value. In Ghana sesame growers rely on wild or undocumented planting seeds which are of mixed colouration and its lignan content is ambiguous. The objective of this study was to segregate street sesame seeds into component colour fractions and subsequently evaluate the consistency of lignans in the seed fractions. Sesame seeds were collected from street vendors in northern Ghana and were segregated into seed fractions based on the pericarp colour. The viability of seed fractions stored at different temperatures (ambient, 5 °C, 0 °C) over time and lignan contents of single or bulk mixed seeds were verified. The collected seeds were of mixed colouration with approximately 4 % debris, 40 % white, 36 % cream, and 20 % dark coloured seeds by weight. The viability of the seeds was 67–85 % depending on pericarp colour. White seed fractions which had the highest proportion by weight had a significantly (p = 0.0275) higher viability (85 %) than the dark seeds which had the least viability (67 %). The seeds lost viability over time. However, seeds stored at 0 °C maintained a viability of 77 % at the fourth year. There were differences in the relative abundance of lignans for both bulk seeds and, single seeds with different pericarp colours. The most abundant occurring lignans in the seeds were sesamin, sesamolin and sesaminol and its downstream glucosides. The source of seed or pericarp colour was not predictive of seed viability or lignan composition. These findings provide baseline data on seed quality including an improved storability under cold environments. It also gives an insight into how mixed seeds of variable pericarp colours can have distinct characteristics. Although the mixed coloured seeds had no implications for varied quality including lignan content, the international market for sesame requires that seeds are of consistent colour.

## 1 Introduction

The emerging preference for natural resources including underutilised crops for dietary requirements has increased the importance and utilization of sesame seed and its oil in medical and food industries [1]. Sesame (*Sesamum indicum*) is an affordable source of protein that is incorporated into food formulations to enhance nutritional quality [2]. Sesame is widely cultivated in many countries in the tropics for its highly prized oilseed. Tropical Africa is considered to be its primary centre of origin where most of the wild sesame species are found [3]. Tanzania for example has replaced India as the leading producer of sesame seeds [4]. There are several improved variants of sesame which are cultivated worldwide or are kept in seed banks. Sesame landraces which include locally adopted varieties, show conspicuous morphological, physiological, and phytochemical diversity, as a result of selection by indigenous farmers or users and also due to repeated domestication over time [5]. The seed or pericarp may be white, cream, beige, tan, yellow, brown, red, grey, black, or even mottled. The general nutty taste and aroma of sesame offer several choices for food enthusiasts. It is used in numerous applications, which is fuelling the industry's expansion. The sesame market size is estimated to reach USD 7.94 billion by 2030 at a compound annual growth rate of 2.1 % [6].

In the commercial seed market, seeds or grains are segregated into high quality (accept fraction), and the low-quality (reject fraction) [7]. The standard sesame seed quality parameters for international trade, include a minimum 98 % cleanliness, 51 % oil content, 6 % moisture, and a maximum of 1 % foreign matter or 2 % off-colour seeds [8]. White bold seeds with high oil content, or the medicinal varieties which have bold black seeds with high oil content also attract higher market price. In the food industry sesame is used as a meal, paste, confections, and in bakery products. Sesame varieties which are stable at high temperatures and able to retain its oxidative properties under baking heat are preferable [9].

Sesame is a source of nutritionally important natural antioxidant lignans such as sesamin, sesamolin, sesaminol and sesamol [10] which are known to possess clinical properties [11,12]. Sesame lignans and especially synthetically available sesamol have also been used as potentiators or synergists of insecticides [13]. Sesamin and sesamolin are the major phenolic compounds found only in sesame plants. The total content of these two lignans in sesame seeds vary to a maximum of 1.4 % [[13], [14], [15]]. The minor lignans present are however in low concentrations and/or are normally by-products of chemical transformations during sesame seed and oil processing. Sesaminol for example occurs in sesame seeds mainly in glycosylated forms [16].

Several solvents have been used for the extraction of lignans for analytical purposes [17]. Differences in lignan content among varieties and accessions have been documented in dozens of studies [18] where the effects of a wide range of agronomic characteristics were used as the causal index of the differences. There have however been contrasting reports on the characteristic effect of sesame seed colour on lignan content. There are however some reported correlations between the content of sesamin and sesamolin [19,20] in sesame seeds which has been used for predictions. The low relationship between the geographical origin and lignan content has been attributed to the long history of intercontinental seed trade [21] which has led to genetic mixtures. The associations reported in some studies may be linked to genetic diversity in the germplasm collections used for such comparisons [22,23].

It is generally known that sesame seeds have lignans however the ratios of the different lignans in sesame for each geographic location is not exhaustively studied. Sesame seeds found in Ghana are of mixed colour although the lighter seed colours are dominant in the mixtures. These seeds are either imported from the Sahelian countries or are locally grown in the northern regions of Ghana. Farmers in Ghana have an ultimate aim to produce white sesame seeds, but the outcomes are inconsistent because, the planting seeds are not certified. There has been reports of increased interest in sesame cultivation in northern Ghana, to the extent that in one district it became a competitor to the staple maize crop for land space. Farmers sow only white seeds but do harvest mixed coloured seeds which is believed to be due to growing and harvesting conditions. The objective of this study was to segregate street sesame seeds into component colour fractions, test the viability and or storability of the fractions under varying temperatures and subsequently evaluate the consistency of lignans in the seed fractions. The results provide the baseline characteristics of a new crop, sesame, in Ghana including its quality, storability, and relative abundance ratios of lignans. These confirm the comparative attributes of mixed coloured sesame seeds sold on the open market which are not predictive of quality.

## 2 Materials and methods

### 2.1 Seed collection and separation

**Fig. 1:**
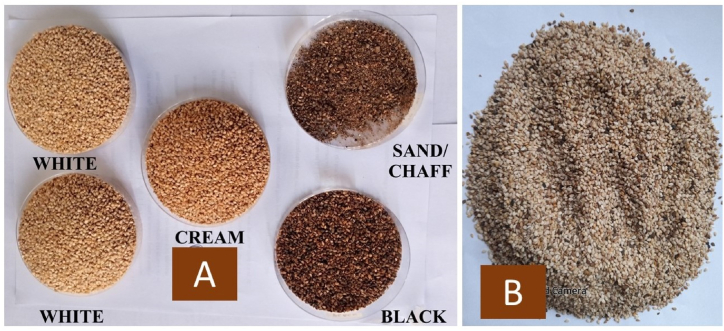
Sample (2 kg) of mixed coloured street sesame seeds sold in northern Ghana. Separated seed fractions from: Tamale/Kpembe-**A**; Sample bulk seeds from Tamale/Kpembe-**B**.

Sesame seeds were randomly collected from open market vendors of Tamale (9.4034° N, 0.8424° W) and Kpembe (8.5320° N, 0.4963° W) in northern Ghana. Seed samples were bulked (Fig. 1B) for each year and manually cleaned by using seed tray to separate them into fractions based on pericarp colour and chaff (Fig. 1A). The separated chaff was discarded whilst the intact seeds were further cleaned of dirt by water floatation. Seeds were then air dried under shade to 6 % moisture and stored at −4 °C until needed.

### 2.2 Viability of white seed fraction under different storage conditions

**Fig. 2:**
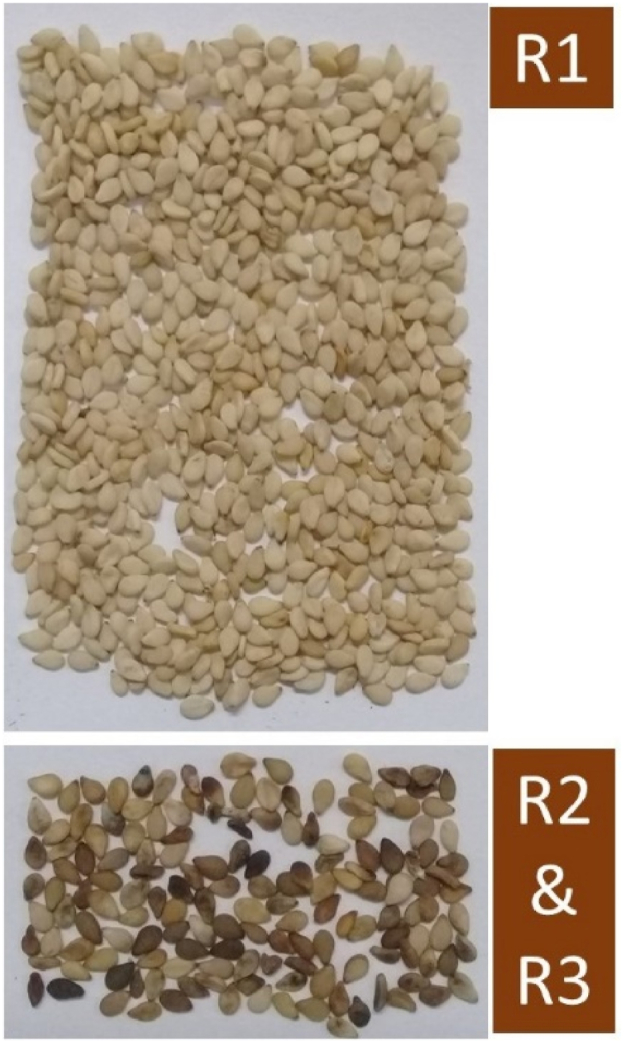
Sample (500g) fractions of sesame seeds from a collected seed mixture. R1 fraction (white) is the preferred fraction by farmers was used for seed viability tests over time. R2/R3 non-white fractions were separated in subsequent collections for seed storability at time zero and lignan content analysis.

Farmers in Ghana do not have access to certified sesame seeds. They rely on self-seed cleaning by selecting for only white pericarp seeds as planting material. Seed samples collected in 2019, R1 (Fig. 2) used for seed longevity tests were stored at either 0 °C, −4 °C or on laboratory shelves (ambient) for four years and these were tested for viability over time (2019–2022). One hundred white seeds were sown in 11 cm petri dishes on paper towels moistened with plain water following LeVan et al. [24]. The set-up was covered and placed on laboratory benches. This was inspected on a daily basis to avoid desiccation. Germination counts were conducted after four days when the radicle had emerged beyond the length of the seed. Seedling elongation after 10 days when green leaves were visible was the index for vigour. The seed viability was evaluated as a percentage of the initial 100 seeds for twelve replicates.

### 2.3 Test for coloured fractions in seed mixture for viability

**Fig. 3:**
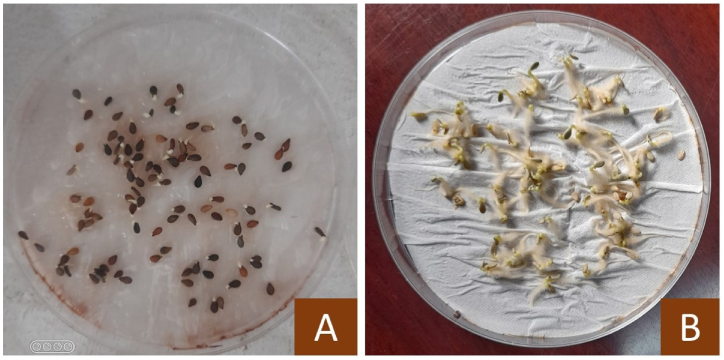
Seed germination test on plain water. (A) After4 days, (B) After 10 days. seed viability was evaluated as a percentage of 100 seeds for12 replicates.

Three coloured fractions of sesame seeds from a freshly collected seed mixture in 2022, (white, cream, dark) obtained by manual separation were tested for germination (Fig. 3A) and viability (Fig. 3B). Standard germination tests were performed on fifteen freshly collected sub-samples of 100 seeds for each pericarp colour fraction.

### 2.4 Lignan extraction and analysis

The difference in the distribution of sesamin, sesamolin, and sesaminol glucosides components in representative samples of sesame were compared from relative abundance of HPLC chromatograms. Fractions from either bulk sample (10 seeds) or single seeds (which was adequate to visualize the limits of detection or quantitation) were subjected to lignan extraction. Briefly, individual, or bulk seeds were transferred into 2 ml tubes containing a Zirconia bead and ground to powder under liquid nitrogen for 20 s. The seed powder was dissolved in 200 μl of 80 % MeOH [25] which extracted glycosides as well. Unlike Ryu et al. [26], crushed seeds were not defatted prior to extraction. The extract was then centrifuged at 21,000×*g* for 5 min after which the supernatant was filtered with Millex-LH (Waters). The filtrate was subjected to HPLC analysis using Cortecs C18+ with Vanguard column 2.7x75 mm. The eluted/mobile phase consisted of water (solvent) and acetonitrile (0.1 % TFA) as described by Rangkadilok et al. [27], with slight modifications. Aliquot samples were each injected and monitored at 2.5 min/sample. Operating conditions were as follows: column temperature 25 °C, injection volume 2 μl and detection at 280 nm. The lignan reference standards were purchased from Nagara Science, Japan, http://www.nsgifu.jp/products/pdf/NGR_sesame201302.pdf.

### 2.5 Data analysis

The weights of the different seed colour fractions were determined as a percentage of the bulk seeds purchased. Differences between the viability of seed colour fractions were subjected to ANOVA and significant means were separated. Germination of stored seeds over a four-year period was expressed as a percentage. Lignan contents are presented as relative abundance (AU) at the retention times.

## 3 Results

### 3.1 Seed fractions and debris

**Fig. 4:**
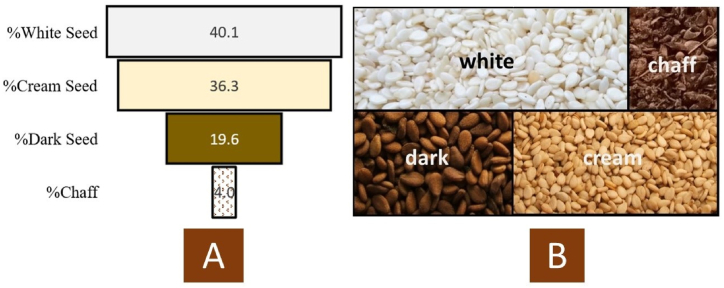
Illustration of seed colour ratios of 2 kg street sesame: (A) Percentage vs (B) Seed Coat (not to scale).

The seeds were separated into four fractions namely white, cream, dark brown and, the chaff portion (4 %) was weighed and discarded (Fig. 4B). The chaff consisted of seed hulls, soil and other plant parts. White seed was 40.1 %, cream seed was 36.3 % and the dark brown seeds comprised 19.6 % by weight (Fig. 4A).

### 3.2 Viability of coloured seed fractions

**Fig. 5:**
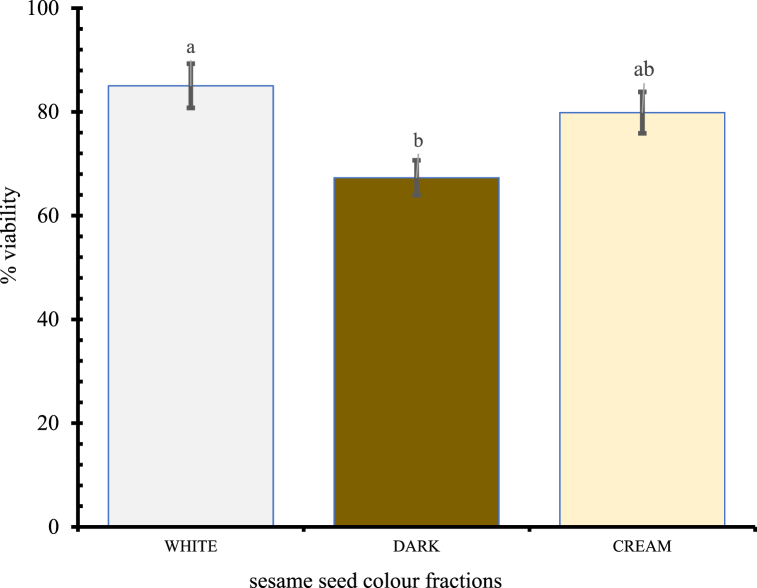
Illustration of varied seed colours of street sesame for percent viability. Plots are with **±5 %** errors, Bars with same letters (a/b) are not significantly different (P = 0.05); seed viability was evaluated as a percentage of the initial 100 seeds for12 replicates.

The intact portion (96 %) of the collected bulk seed was subjected to germination test two weeks after collection from the open market. The results indicate that viability of white seeds (85 %) was significantly higher (P = 0.02346) than the dark brown seeds (67.3 %) (Fig. 5).

### 3.3 Viability of white seeds under storage

**Fig. 6:**
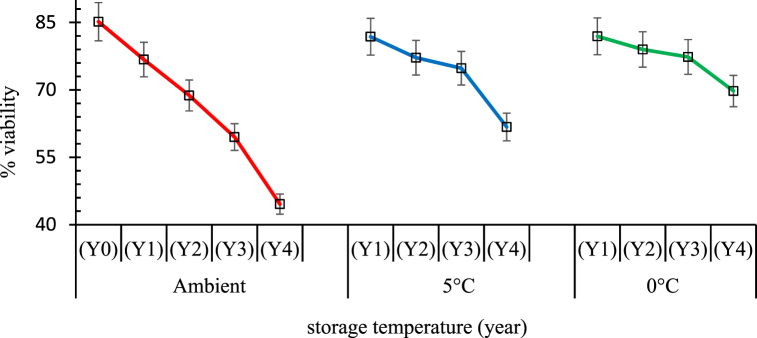
Illustration of stored white seeds for viability under-freezing (0 °C), cold (5 °C) and ambient (∼27 °C) temperatures over four years (Y1-Y4). Plots are with **±5 %** errors for each storage condition; seed viability was evaluated as a percentage of 100 seeds for12 replicates.

There was a general decline in seed viability over time (Fig. 6). White seeds kept at ambient conditions on laboratory shelves had a viability of up to 45 % at the 4th year. Storage conditions in a kitchen fridge (5 °C) led to a viability decline from 77 % at year one to 62 % at year four. Seeds stored in a freezer (0 °C) maintained a viability of 70 % by the fourth year.

### 3.4 Lignan profile of bulk seeds from two locations

**Fig. 7:**
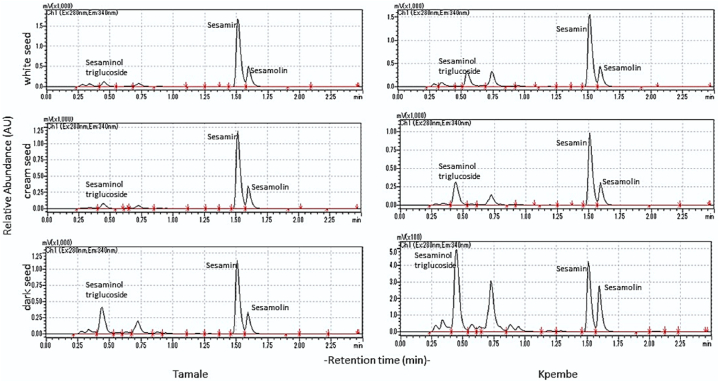
Sample lignan profiles from bulk seeds collected from two locations Kpembe/Tamale. Fractions of coloured seeds from either bulk sample (10 seeds) or single seeds.

The relative abundance of sesamin, sesamolin and sesaminol determined for seeds collected from two sites in Ghana, Tamale and Kpembe had inconsistent intensity of refraction index signals from the chromatograms (Fig. 7). Generally, presence of sesamin and sesamolin contents in all seed colours were not predictable. The presence of sesaminol was higher in seeds collected from Tamale (300–500 AU) than that from Kpembe, which was 100–350 absorbance units. The relative abundance of lignans from the seed fractions between Kpembe and Tamale respectively were 1500 vs1500 AU (white seed), 1250 vs 1000 AU (cream seed) and 1250 vs 500 AU (dark seed). Seeds from Tamale however contained Sesaminol triglucoside regardless of the pericarp colour unlike that from Kpembe where cream seeds had very low sesaminol.

### 3.5 Lignan profile from single white seeds

**Fig. 8:**
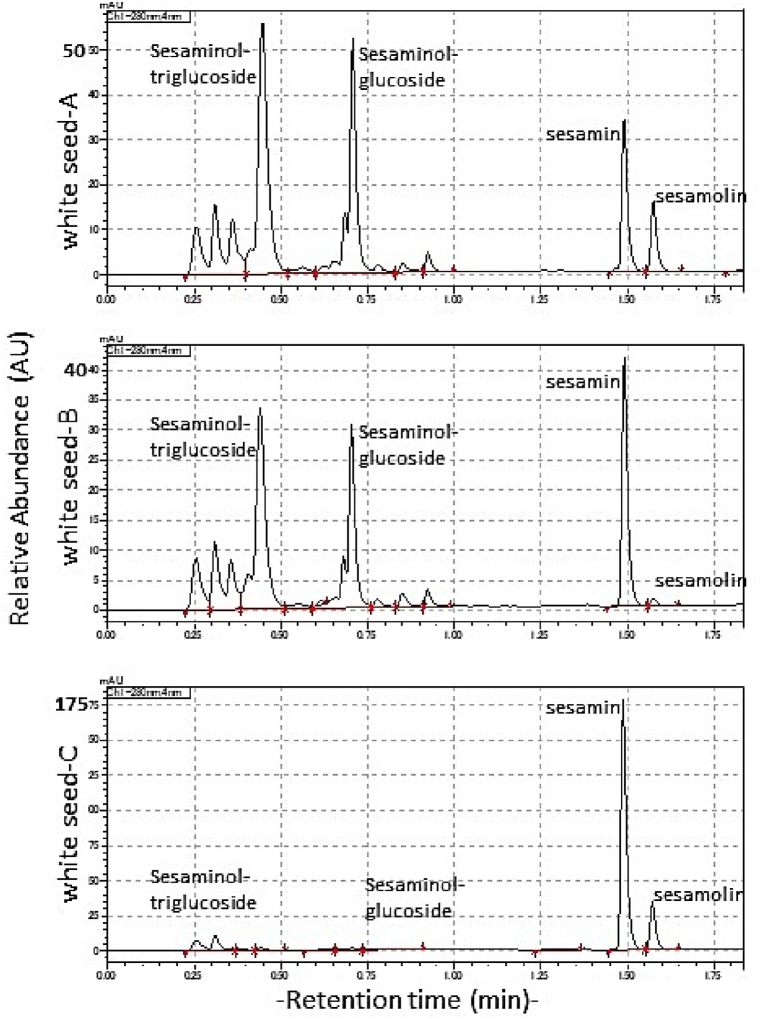
Sample chromatograms showing non-uniform lignan profiles of single white seeds from same location, Kpembe (Ghana). Sample chromatograms from 10 individual coloured seeds A, B, C.

Results from single white seeds from Kpembe tested for lignan contents by HPLC indicated that sesamin was always present in a seed (40–175 mAU), but the other lignans were not consistent (Fig. 8). For example, the chromatogram for white seed C, showed that the level of sesaminol triglucoside, was under detection level. The ratio of sesamin and sesamolin was seed specific with no predictability in these white seeds.

### 3.6 Lignan profile from single white vs brown seeds

**Fig. 9:**
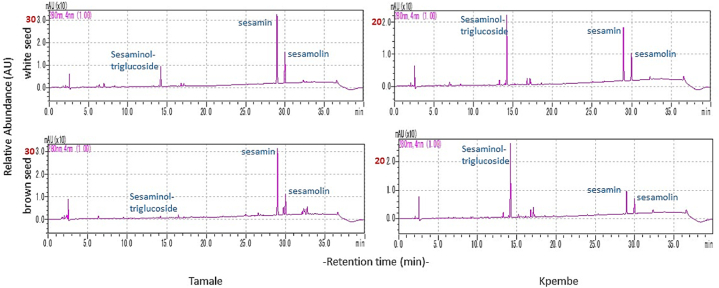
Comparative lignan profiles of single white vs brown sesame seeds from two sampling sites, Tamale and Kpembe. Sample chromatograms out of 10 individual coloured seeds.

Lignan profiles of single white or brown sesame seeds from two sampling sites, Tamale and Kpembe had a maximum lignan relative abundance of 30mAU for Tamale seeds, and 20 mAU for Kpembe seeds irrespective of pericarp colour. For single seeds irrespective of pericarp colour, sesamin and sesamolin was present (Fig. 9).

## 4 Discussion

Although sesame production is gradually gaining recognition in Africa as depicted in the increased area under cultivation [28], it is still a neglected crop in many countries including Ghana. In most countries there are no commercial varieties available for cultivation and there is limited conscious national research programs where many refer to as an orphan crop. Demand is on the rise for sesame seeds worldwide however, supplies are running low due to quality challenges. The availability of quality sesame seed is vital and crucial for sustainable production. Access to good sesame seed is one of the key criteria needed to help boost production and trading both locally and on the international market. Farmers especially in Africa therefore grow landraces whose seeds are exchanged and recycled for generations as done for bambara groundnut or fonio [29,30]. The quality of such recycled seeds is either low, variable or are unknown [31]. Seed quality for most edible seeds and grains is well known to differ based on seed coat colour [32]. This was not the case for the sesame samples collected from Ghana. Here the quality did not have a predictive relation with seed colour.

To successfully enter the elite sesame market, the product should be of high quality and must fulfil stringent food safety requirements [33]. Therefore, checking seed purity plays a significant role in seed production, and in the general trading of seeds. A good quality sesame seed can be identified by taking a closer look at the appearance for the preferred shape, colour and flavour. Whitish seeds are believed to be of a highest quality for food whilst the black seed is preferred for high oil content. For seed physical dimension, non-flat or bulging and well dried seeds are known to be the highest quality seed.

Harvested seeds usually contain trash and other inert material, weed seeds, deteriorated, and damaged seeds, and off-size seeds. Results from this survey indicated that the chaff content was about 4 %. There is therefore a need for further cleaning before such seeds could enter the commercial market. Seeds sold on the local markets are mostly off-taker seeds with no seed processing apart from minimum drying to safe moisture levels.

Growing mixtures of cultivars as a cost effective measure and diversity is a common practice in many regions of Africa and South America [34]. However, this practice reduces the premium on seeds for international markets where single coloured grains are preferred. Separating seeds for uniqueness, uniformity and stability standards are also required for variety registration, patents, and other quality assessments before new varieties can be accepted in the markets [35]. The results showed that sesame seeds sold in Ghana had a high purity but still fell short of the 98 % purity, which is the minimum recommended standard for international trade. The usable portion of the samples in this experiment (96 %) was a mixture of three coloured seeds, which is not desirable on the international market. The chaff portion from the samples which was 4 % is not acceptable as it contained most of the described components including broken and damaged seeds, sand, frass, and other insect body parts.

Certain physical characteristics of sesame namely size, density, and length of seed can be relied upon as indirect qualities for germinability and vigour. These morphological traits are important diagnostic characters when selecting also for seedling vigour. Seed quality is important for agriculture since there are established correlations between good seed and high resistance to both biotic and abiotic stresses, germination rate and plant performance [36]. Quality seeds guarantees high yields leading to high production and subsequent profit margins. Gislum et al. [37], indicated that the concept of seed quality was composed of several attributes, including germination capacity, viability, and uniformity. The presence of weed seeds, diseases and insect infestation has also been included in the standards for seed quality [38] which could impinge on viability.

Viability of stored seeds has widely been reported to be related with storage conditions including temperature and duration [39]. More than 80 % of the area under sesame is still sown with seed saved by farmers from previous harvests. Seeds stored for sowing are usually stored for about 4 years [40,41] which is within the viability range of the seeds stored below 5 °C in the sesame seeds tested. Farmer saved seeds which are mostly of mixed varieties may not necessarily be of quality but performance-wise, seed mixtures may comprise different varieties with different maturity periods, yields and responses to environmental conditions or resistance to biotic stressors that make them versatile.

Seed damage may also occur during harvesting, cleaning, storage, and processing or before harvest from disease infestation or insect activities on the field or in store. These damage may be presented as cracks in the seed coat and splitting of the seed which subsequently leads to losses in germination and vigour [42]. The physiological quality of a seed typically comprises its germination and vigour [7]. The tested stored seeds under different temperatures and time were necessary to determine the effect of ageing [43] or temperature [44] on seed quality. Although Silva and Cicero [45] and Wulansari et al. [46], have reported that germination tests usually overestimate the quality of seeds since such tests are done under optimum conditions, data on percentage of dormant and germinating seed is still required for labelling seed packages for sale [47].

To test for seed dormancy some authors adopt accelerated aging where seeds are exposed to high temperatures and relative humidity for a short period of time. However, in this experiment, the seeds were rather stored at different temperatures over a four year period using the aging-driven seed longevity as described by De Vitis et al. [48], and also reported by Solberg et al., [49]. The use of pericarp colour in this experiment to discriminate viability was informed by Reed et al. [50], that seed performance is expected to be affected differently by seed colours. Our results however add to the inconsistency or unreliability on seed coat colour as a quality index for germination. Lighter seeds from our seed mixture had a higher viability than dark seeds. A plausible explanation could be that discolouration to dark seeds were not inherent seed characters but rather were immature, damaged, or diseased since earlier reports have indicated elsewhere that, black sesame seeds can have high germination [51]. Conditions during seed maturation, harvesting and storage can also influence the colouration of seeds. In conformity with Desheva [52], seeds stored at cold temperatures had a higher viability within the four years when this experiment was terminated. However, by the fourth year, seed viability was above the half-life for the tested white seed fraction which were stored under freezing conditions. Seed longevity studies by Singh et al. [53], confirms the influence of low temperature and initial moisture content of sesame seeds, where at ambient conditions seeds were viable for only one year but could be greater than 86 % if stored at −18 °C coupled with prior drying to an equilibrium at 15 % RH.

Generally, plant tissue is an expression of the genomic, physiological, biochemical, and environmental factors under play. Seed coat colour has been reported as a crucial trait in sesame with a linkage to the phytochemical composition of seeds [23,54] and subsequent biochemical properties [55]. Seed colours of sesame is known to affect the phytochemical contents and their biological activities [56]. Other studies have indicated that seed coat colour sometimes interfere with antioxidant and lignans in sesame seeds and was linked to abiotic stressors [57]. The Lignan profiles of bulk seeds from the two locations surveyed in this experiment were variable. Although the longest distance between the collection sites was about 40 km, factors such as agronomic practices, biotic stressors, harvesting and post-harvest processes could contribute to the variations in the lignan compositions. Sesamin, sesamolin and sesaminol triglucoside were present at varying relative abundance in each colour of bulk seeds tested from the two locations.

The abundance of phytochemicals in sesame in relation to seed coat colour is highly debatable with no conclusive winner. Light-coloured sesame seeds have been reported to contain higher levels of polyphenols and are richer in lignans than coloured sesame seeds [22,58,59]). However, an evaluation by Wang et al. [60], put brown seed coat, as highest store of sesamin and sesamolin which was similar to the reports by Ajit et al. [15], or Shi et al. [18], that dark seeds had higher lignan contents than lighter seeds and ranked them as yellow > black > brown > white in order of decreasing lignan content. This is similar to the report that phytochemicals in sesame seeds was correlated with seed coat colour from white to dark variations through intermediate colours [61,62]. Lignan profile from single white seeds collected from the two locations in northern Ghana had no consistency. Random white or brown seeds collected from the same location had variable lignan contents. Although sesamin and sesamolin were constantly present in all seeds in inconsistent proportions, sesaminol and its downstream metabolites, especially sesaminol glucosides, was suspected to respond to changes in local biotic and abiotic factors including seed maturity, water deficit and pest damage which need to be verified.

These inconsistencies may be similar to other reports where specific lignans were higher for different seeds. For example, Chellamuthu et al. [63], indicated that white sesame seeds had higher sesamol content whereas black varieties contained higher sesamin. It must be noted however that sesamol is usually a degradation product of heated sesame either of the seed or oil and occurs in trace amounts in raw seeds [13,64]. This study becomes part of the debate on whether seed colour and lignan content in sesame are related since others [23,60] have discounted the existence of a correlation between lignans and seed colour and rather associated it with the specific germplasm used in studies. The differences in sesame attributes due to geographic locations as used in this study as a source of variation has also been discounted by Pathak et al. [21], since they believed that such variations should be expected because sesame was an old crop whose seed has been exchanged extensively even at intercontinental levels.

## 5 Conclusion

This study has provided critical baseline data for sesame in a country where it is a new and emerging crop with no research activity. It is indicative that sesame culture is still underdeveloped in Ghana and actors in the trade could be considered as ephemerals who are lurching on a perceived profitable venture with hindsight from neighbouring countries. This is because the sesame trade is less regulated, and quality is highly compromised in lieu of the level of chaff found in the Ghanaian samples studied. In the absence of structured research leading to the provision of certified seeds, farmers store their seeds using traditional holding containers, which has been found to be unsustainable. Results here reveal that storing sesame seeds at low temperatures can serve as a safe and relatively inexpensive method of prolonging the longevity or viability of sesame seeds. This was tested using either household fridges or freezers which kept viability for at least four years which is adequate for local farmers without access to certified seeds. Relative lignan content from the coloured seeds collected in this geographic region varied over a wide range, which is similar to other results reported in literature. The relative lignan contents of sesame seeds are related though inconsistent with pericarp colour. In most cases relative sesamin presence was correlated with sesamolin but at unique proportions for each individual seed. Although seed coat colour did not influence lignan quality, the preference for uniform seeds especially of white colour on the market is undeniable. Further sorting of the seeds by colour could be a competitive edge for farmers to meet international standards, whilst the off-coloured seeds are sold locally and could be incorporated in animal rations due to the high protein content of sesame or extracted for its quality oil.

## Ethics approval and consent to participate

Not applicable.

## Consent for publication

The author has the sole responsibility for the publication.

## Availability of data and materials

The data generated in this study has been deposited in the public repository, figshare and it's available 10.6084/m9.figshare.26388634.

## Competing interests

The author declares that no known competing financial interests or personal relationships that could have appeared to influence the work reported in this paper.

## Author contribution

Henry O. Sintim designed the research study, performed the research, analysed the data, wrote the manuscript, made the editorial changes in the manuscript and approved the final manuscript.

## Funding

This research was partially funded with the personal institutional annual book and research stipend allocated to the author. However, this work had no bespoke grant that supported the activities. Suntory Foundation for Life Sciences: Seika, Kyoto, Japan sponsored and performed the biochemical analysis in kind.

## Declaration of competing interest

The authors declare that they have no known competing financial interests or personal relationships that could have appeared to influence the work reported in this paper.
